# An *In vivo* Multi-Modal Structural Template for Neonatal Piglets Using High Angular Resolution and Population-Based Whole-Brain Tractography

**DOI:** 10.3389/fnana.2016.00092

**Published:** 2016-09-27

**Authors:** Jidan Zhong, David Q. Chen, Matthew Walker, Adam Waspe, Thomas Looi, Karolina Piorkowska, James M. Drake, Mojgan Hodaie

**Affiliations:** ^1^Division of Brain, Imaging and Behaviour – Systems Neuroscience, Krembil Research Institute, University Health Network, TorontoON, Canada; ^2^Institute of Medical Science, University of Toronto, TorontoON, Canada; ^3^Centre for Image Guided Innovation and Therapeutic Intervention, The Hospital for Sick Children, TorontoON, Canada; ^4^Division of Neurosurgery, The Hospital for Sick Children, TorontoON, Canada; ^5^Division of Neurosurgery, Toronto Western Hospital – University of Toronto, TorontoON, Canada

**Keywords:** neonatal piglet, template, multi-modal, diffusion imaging, population-based whole-brain fiber tracts

## Abstract

An increasing number of applications use the postnatal piglet model in neuroimaging studies, however, these are based primarily on T1 weighted image templates. There is a growing need for a multimodal structural brain template for a comprehensive depiction of the piglet brain, particularly given the growing applications of diffusion weighted imaging for characterizing tissue microstructures and white matter organization. In this study, we present the first multimodal piglet structural brain template which includes a T1 weighted image with tissue segmentation probability maps, diffusion weighted metric templates with multiple diffusivity maps, and population-based whole-brain fiber tracts for postnatal piglets. These maps provide information about the integrity of white matter that is not available in T1 images alone. The availability of this diffusion weighted metric template will contribute to the structural imaging analysis of the postnatal piglet brain, especially models that are designed for the study of white matter diseases. Furthermore, the population-based whole-brain fiber tracts permit researchers to visualize the white matter connections in the piglet brain across subjects, guiding the delineation of a specific white matter region for structural analysis where current diffusion data is lacking. Researchers are able to augment the tracts by merging tracts from their own data to the population-based fiber tracts and thus improve the confidence of the population-wise fiber distribution.

## Introduction

The domestic piglet is a common animal model for translational research in pediatric neuroscience because of its neuroanatomical commonalities with the human brain (Lind et al., 2007; Sauleau et al., 2009; Mendl et al., 2010; Gieling et al., 2011), with respect to the morphological shape of the piglet brain including the gyral and sulcal pattern, and the rapid period of brain growth lasting from late prenatal to early postnatal (Dickerson and Dobbing, 1967; Thibault and Margulies, 1998). There is an additional advantage in that the piglet brain is of sufficient size to allow *in vivo* multimodal imaging and facilitates surgical procedures and electrode placement (Sauleau et al., 2009).

Previous imaging research based on imaging analysis of multiple piglets has required manual evaluation at the individual level due to lack of a common template (Conrad et al., 2012; Radlowski et al., 2014). Conrad et al. (2012) performed intensive manual segmentation of individual brain regions of interest (ROIs) over seven longitudinal scans of 15 piglets. Saikali et al. (2010) published a high-resolution surface-based three-dimensional (3D) digital template of a female pig (Sus scrofa domesticus), segmented into 178 cerebral structures. This template proved useful for localizing brain areas for the purposes of functional magnetic resonance imaging (MRI) studies or electrode implantation trials. However, the template was constructed based on a single brain of a 6-month old pig. This creates important limitations in its use since age results in a significant difference in the total brain volume (TBV) as well as the proportion of cortical volume over TBV in pigs (Conrad et al., 2012). In order to address this deficiency, two additional templates have been published recently, based on T1 images from neonatal piglets. Conrad et al. (2014) generated an *in vivo* 3D T1 template of the neonatal piglet (4-week old, Sus scrofa). Still, this template lacks adequate sulcal and gyral information because linear registration was performed to generate the template and non-linear deformation fields were only applied to compensate for template shape or morphology. Gan et al. (2014) generated a T1 template based on a group of neonatal piglets (38 days old, Suscrofa × Landrace × Large White F1). The Gan template shows improved gray matter/white matter (GM/WM) contrast, however, important anatomical areas such as the optic chiasm and bulbus olfactorius needed to be removed to allow for better positional accuracy, limiting the usability of this template. Importantly, both the Conrad and Gan templates provide very limited identification of specific WM tracts, since they are based on T1 images which provide a homogeneous WM appearance (Toga et al., 2006).

The limitations of the existing templates clarify the need for a multi-modal template for the neonatal pig that includes information on WM tracts. The most advantageous technique that permits information on WM tracts is diffusion weighted MR imaging (DWI). This technique non-invasively measures the diffusivity of water molecules and characterizes the integrity of white matter fibers within the tissue based on diffusion orientation information (Basser et al., 1994). By modeling the directional diffusion of water as an ellipsoid, or “tensor”, quantitative information is provided, such as axial diffusivity (AD), radial diffusivity (RD; Song et al., 2003), mean diffusivity (MD; Cercignani et al., 2000) and fractional anisotropy (FA; Basser and Pierpaoli, 1996). With this technique, white matter has been shown to be implicated in children and adolescents in a wide range of disorders, including autism spectrum disorder (Mak-Fan et al., 2013; Conti et al., 2015), attention deficit hyperactivity disorder (ADHD; Lawrence et al., 2013) and Turner syndrome (Villalon-Reina et al., 2013). Other than quantitative measures, diffusion tractography derived from DWI is also a powerful measure to aid image interpretation through visualization of the orientation and 3D course of white matter tracts (Hiltunen et al., 2005). It helps clarify the architecture of tissues by integrating the estimates of voxel-based diffusion maximums (Feldman et al., 2010; Alexander et al., 2011). It also allows visualization of specific fiber connections and comparison across individuals (Witwer et al., 2002; Yeatman et al., 2009), definition of specific ROIs (Schlaier et al., 2015), as well as characterization of connectivity properties (e.g., fiber density and fiber count) between ROIs (Kim et al., 2011).

There is a clear paucity of literature exploring tractography in the piglet model. A few studies have applied diffusion imaging to detect brain WM alterations in piglets through comparison of FA values, as a consequence of low birth weight (Radlowski et al., 2014), perinatal choline deficiency (Mudd et al., 2015) and dietary treatment (Mudd et al., 2016). Winter et al. (2011) assessed the microstructural changes during pig brain development by measuring MD and FA values. However, none of the studies have reported tractography results. Furthermore, their relatively small number of direction of their diffusion-encoding gradient (≤30) made it hard to resolve the crossing fibers (Tournier et al., 2011). High angular resolution diffusion imaging (HARDI) techniques allow reconstruction of more accurate models for the diffusion process to resolve multiple fiber routes in the same voxel, and thus provide a more accurate WM geometry in the brain (Alexander et al., 2002; Tuch et al., 2002). Thus, tractography that is based on HARDI in the piglet brain allows *in vivo* visualization of WM connections with more accurate presentation of crossing fibers, and can also guide specific ROI delineation and brain anatomy dissection for structural analysis.

Overall, a multi-modal template incorporating T1, DWI metrics and tractography would allow a comprehensive, multi-modal depiction of the piglet brain and serve as an important reference for piglet brain imaging studies. Our present study reports the creation of a multi-modal piglet brain template that includes T1, DWI metric map and population-based whole-brain fiber tracts. Each of the modalities provides an important component to the template. T1 images provide good contrast between GM and WM, and diffusion metrics characterize the integrity of white matter fibers. These metric maps also allow the possibility of advanced automated structural analysis to investigate the WM changes at the voxel-level. Furthermore, the population-based whole-brain fiber tracts can provide valuable information on WM connections across subjects, with fiber reliability and variability estimated through the inter-subject consistency of the fibers.

## Materials and Methods

### Ethics Statement

These experiments were approved by the Animal Care Committee and Lab Animal Services at the Hospital for Sick Children. This study follows the Canadian Council on Animal Care (CCAC) standards.

### Piglet *In vivo* Models

Eight male Yorkshire piglets of 22 ± 6 days old (body weight: 6.1 ± 1.2 kg) were used in this study. All piglets were housed in pairs in a temperature- and humidity-controlled environment with a 12-h light/dark cycle and fed with a commercial piglet milk replacer prior to MRI scanning. The piglets were euthanized by intravenous injection of pentobarbital sodium (120 mg/kg) upon the completion of all imaging procedures and while under general anesthesia.

### Magnetic Resonance Imaging

The animals were pre-anesthetized with a Ketamine (10 mg/kg) solution intramuscularly, intubated and maintained under anesthesia with 2.5% isoflurane and 2 L of oxygen delivered via MRI-compatible ventilator during the MRI scanning. Heart rate and oxygen saturation were monitored and a circulating water blanket was used to maintain core body temperature of 37°C. The animals were placed prone and head first on the diagnostic table. In the Philips Achieva 3T MR scanner, a 32-channel receive-only head coil was placed around the head to provide MR imaging. A three-dimensional T1-weighted magnetization prepared gradient echo (MPRAGE) sequence was used. The sequence parameters were: repetition time (TR) = 8.15 ms, echo time (TE) = 3.72 ms, flip angle = 8°, matrix = 224 × 224, field of view (FOV) = 224 mm × 224 mm, slice thickness = 1.00 mm.

The diffusion-weighted images were collected with a spin-echo single-shot echo-planar imaging (EPI) sequence using sensitivity encoding (SENSE; TR = 5844.97 ms; TE = 105.90 ms; flip angle = 90°; matrix = 128 × 128, FOV = 205 mm × 205 mm; slice thickness/spacing = 1.60/1.60 mm; SENSE reduction factor = 2) with a *b*-value of 800 s/mm^2^ along 128 directions. A baseline image with b = 0 s/mm^2^ (b0 image) was acquired for both forward and reverse phase encoding directions.

### Image Processing

#### T1 Template Generation

To minimize the manual work for the brain mask delineation, template generation was initiated with manual registration of the individual T1 images to the Conrad T1 template to provide a general individual brain mask using 3D Slicer (version 4.4)^1^. The mask was then manually corrected to remove possible remaining non-brain tissue (e.g., extra skull and cerebral spinal fluid (CSF) between the skull and brain). The T1 brain images were then collected to generate a population-averaged template using symmetric diffeomorphic registration (SyN) with Automated Normalization Tools (ANTs; Avants et al., 2011b). The template was then reoriented along a line connecting the centers of the anterior and posterior commissure (y-axis). The origin was set to be the anterior limit of the posterior commissure in the midsagittal plane, consistent with published pig templates (Felix et al., 1999; Watanabe et al., 2001; Saikali et al., 2010; Conrad et al., 2014; Gan et al., 2014). The FMRIB’s Automated Segmentation Tool (FAST) of FSL (Analysis Group, FMRIB, Oxford, UK)^2^ was applied to segment the individual brains into GM, WM, and CSF. The probability map for each segmentation was then transformed to the final template and averaged. To calculate the individual brain volume, the brain mask of the final T1 template was transformed back to individual space based on the transformation field generated during the template generation.

#### Landmark Variation

Distance variations between the individual subject and the template were calculated to validate the template. The anterior and posterior extents of the corpus callosum (CC), and the anterior extent of the posterior commissure were picked as the landmarks. These landmarks were transformed into the template space based on the registration and their spatial locations were compared to the template to compute the landmark variation. Mean (±standard deviation), minimum and maximum distances between individuals and the template in the landmarks were calculated in Matlab (version 2014b, MathWorks, Natick, MA, USA).

#### DWI Process

The DWI sequences were corrected for distortions induced by susceptibility-induced off-resonance field using “Topup” in FSL (Andersson et al., 2003). Following this, the images were aligned to their b0 images with an affine registration to correct for eddy-current and motion distortions using FLIRT in FSL (Jenkinson et al., 2002). Additionally, the gradient vectors were corrected with the appropriate rotational component of the motion correction to ensure that errors in the diffusion weighting that originate from these rotations could be minimized (Leemans and Jones, 2009). The data were resampled for a final voxel size of 1 mm × 1 mm × 1 mm. Diffusion tensors (DTs) were fitted at each voxel to calculate scalar maps: FA, AD, RD, and MD.

To perform whole-brain fiber tracking with the MRtrix software package (Brain Research Institute, Melbourne, VIC, Australia^3^), an intra-subject T1 to DWI space transformation was obtained through manual registration using FA as the feature using 3D Slicer. Individual brain masks were transformed from T1 space to DWI space to be the mask for whole brain tractography.

The response function for a single fiber population was estimated for those voxels with a FA > 0.3 using an iterative optimization method proposed by Tax et al. (2014). The response function was then used for constrained spherical deconvolution to accurately estimate the fiber orientation distribution (FOD; Tournier et al., 2007). Fibers were then generated with a deterministic tracking algorithm, referred as SD-Stream, that follows the orientation of the nearest FOD peak at each step (Tournier et al., 2012). The algorithm generated 10,000 fibers of minimum length 5 mm. Other tracking parameters included a step size of 0.1 mm, minimum radius of curvature of 1 mm and FOD cutoff of 0.2. All voxels in the white matter mask segmented in the previous mentioned step were used as seeds and the tracking procedure was stopped if a fiber reached a voxel outside the mask or if a stopping criterion was met (high fiber curvature or low FOD). To reduce the size of the data, we decreased the density of the points along the length of the streamline by half. Finally, all the scalar values (FA, AD, RD, and MD) were interpolated at each point of the fibers.

Based on the intra-subject registration between T1 and DWI and the inter-subject registration between T1 and the T1 template, all scalar maps were transformed into the template space and averaged to be the corresponding metric template. Using software developed in-house that non-linearly deforms fiber tracts based on the registration transformations, the individual whole-brain fiber tracts were transformed into the template space and merged as population-based whole-brain fiber tracts. The scalar information was also kept on the population fiber tracts.

### Application: ROI-Specific Fiber Tracts

Specific ROI-based fiber tracts were extracted from the population-based whole-brain fiber tracts. The ROIs include genu and splenium of the CC, optic chiasm, fornix and middle cerebellar peduncle. 3D Slicer was used to extract the fibers passing through specific fibers.

### Statistical Analysis

Mean (±standard deviation) body weight, age and brain size of the piglets used to generate the templates were calculated in Matlab (version 2014b, MathWorks, Natick, MA, USA).

## Results

### Animal Modal Parameters

The average age of the eight piglets was 22 days (range: 17∼35 days old). The average brain size of the piglets was 65500 ± 3806 mm^3^, and the template brain size was 67294 mm^3^. For the six pigs used to generate the DWI related template, the average body weight, age, and brain size were 6.5 ± 1.2 kg, 23 ± 6 days old and 66434 ± 3927 mm^3^. The body weight, age and brain size of the eight piglets used to generate T1 template are shown in **Table 1**.

**Table 1:** Body weight, age and brain size information of the piglets used for T1 template and DWI template generation.

Pig index	Body weight (kg)	Age (days)	Brain size (mm^3^)	Template generation
1	4.9	17	61606	T1
2	5.0	19	63787	T1
3	5.1	18	62279	T1, DWI
4	8.3	35	73946	T1, DWI
5	6.7	21	65359	T1, DWI
6	7.1	21	68884	T1, DWI
7	5.3	21	63573	T1, DWI
8	6.3	21	64563	T1, DWI
Avg for T1	6.1 ± 1.2	22 ± 6	65500 ± 3806	–
Avg for DWI	6.5 ± 1.2	23 ± 6	66434 ± 3927	–

*Avg for T1- Average of the demographic information over the pigs used to generate the T1 template; Avg for DWI- Average of the demographic information over the pigs used to generate the diffusion related template.*

### T1 Template and Segmentation

**FIGURE 1:**
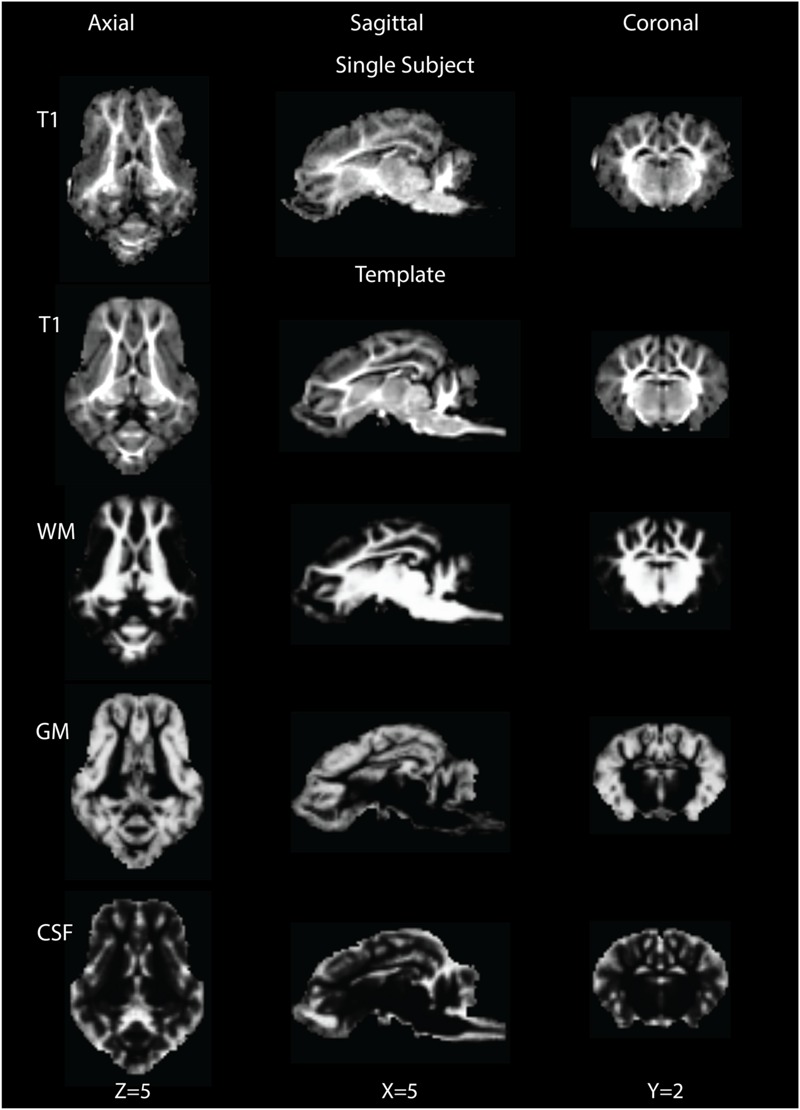
**Single subject T1 image (row 1) and template T1 image (row 2) are shown in the axial (*Z* = 5), sagittal (*X* = 5) and coronal (*Y* = 2) views.** Rows 3∼5 show tissue probability maps for white matter (WM), gray matter (GM) and cerebrospinal fluid (CSF) in the template space.

The brain pattern is well preserved in the final T1 template brain compared to that of a single subject T1 image (**Figure 1**, row 1). The final T1 template brain is shown in **Figure 1** (row 2) in the axial, coronal and sagittal views. Qualitatively, the outline of brain structures and GM/WM contrast are clearer in the template than in the single subject. Based on the intensity differences, the T1 brain image could be segmented into GM, WM and CSF. **Figure 1**, rows 3–5 shows the probability maps of GM, WM and CSF in the template space.

### T1 Template Validation

The maximum distance was 1.73 mm for one subject at the posterior CC, while the mean distances across subjects were smaller than 1 mm for all three landmarks. The differences between coordinates in selected landmarks are shown in **Table 2**.

**Table 2:** The distance differences between individuals and the template in selected landmarks.

	Distance, mm (mean ± SD)	Max distance, mm	Min distance, mm
Anterior CC	0.85 ± 0.52	1.41	0
Posterior CC	0.72 ± 0.60	1.73	0
Anterior end of posterior commissure	0.50 ± 0.50	1.00	0

### DWI Template

**FIGURE 2:**
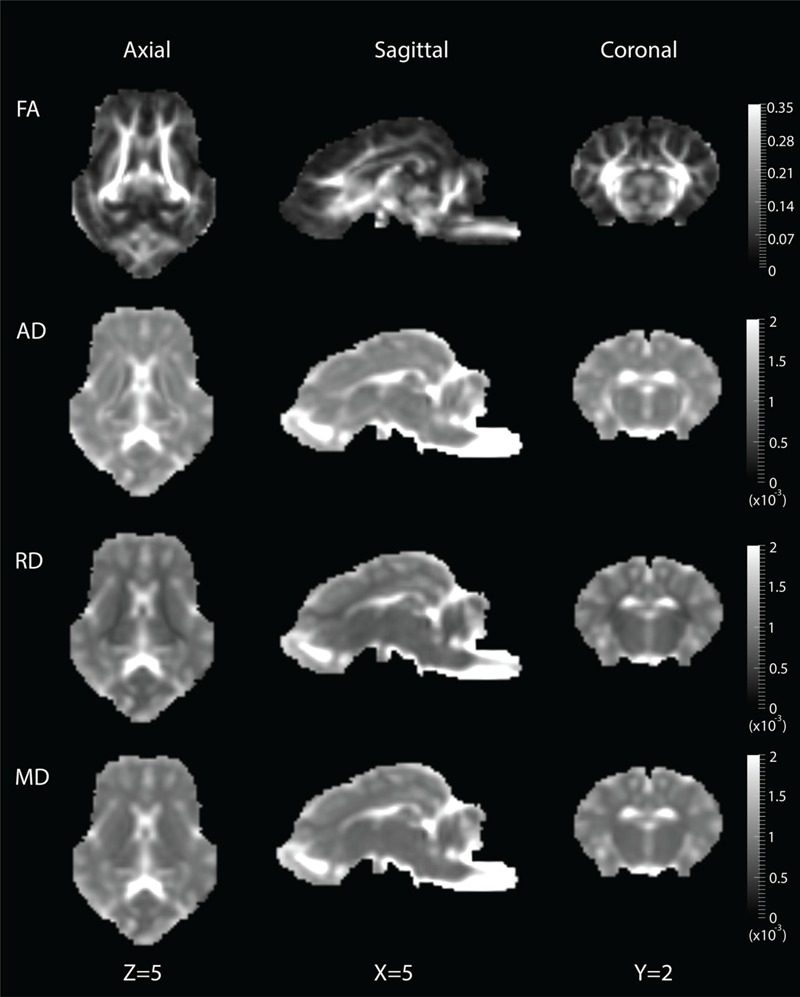
**Diffusion metric templates with FA, AD, RD and MD maps are shown in the axial (*Z* = 5), sagittal (*X* = 5) and coronal (*Y* = 2) views**.

**FIGURE 3:**
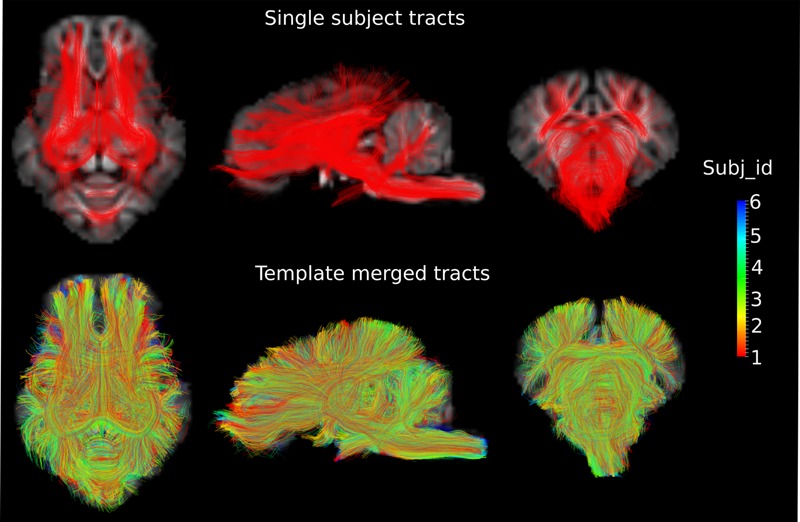
**Fiber tracts from a single subject and a group of subjects are shown in the axial (*Z* = 3), sagittal (*X* = 0) and coronal (*Y* = -1) views in the template space, with the template T1 image as the background.** Different colors represent the fibers from different individuals.

**FIGURE 4:**
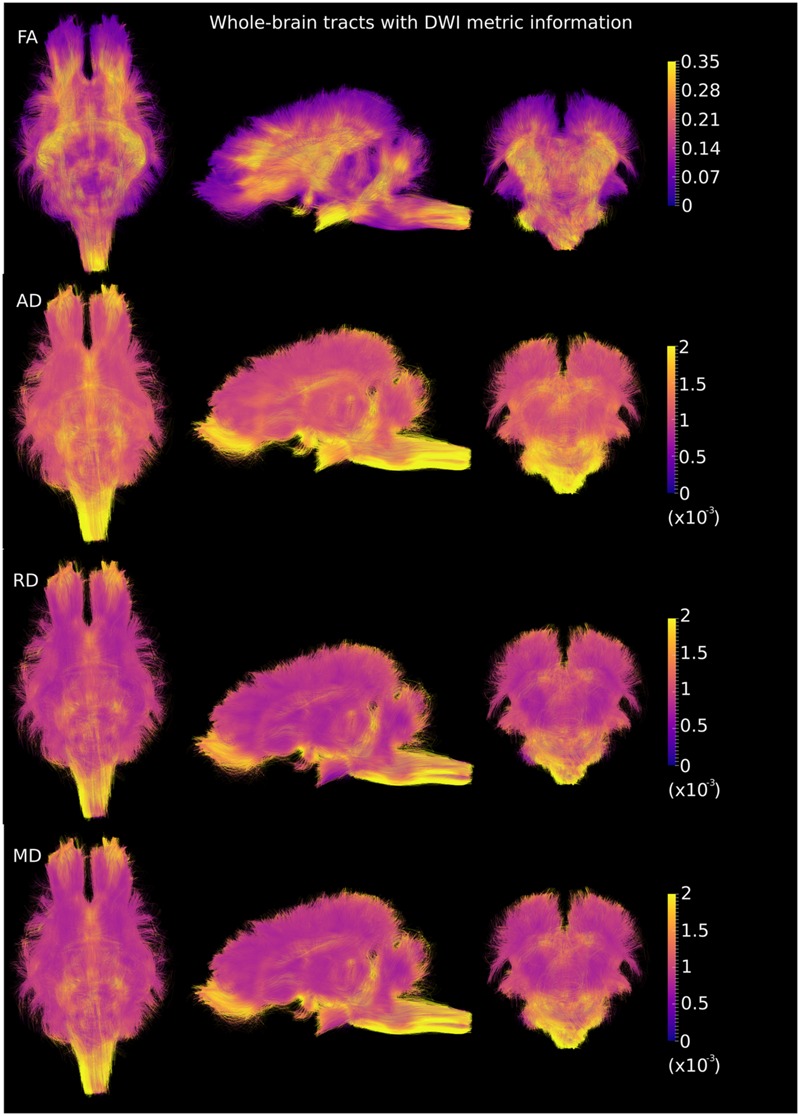
**Merged whole-brain fiber tracts overlaid with DWI metrics (with the same value scale as in **Figure 2**) in the axial, sagittal and coronal views.** Brighter color represents higher values.

DWI metric templates, including FA, AD, RD, and MD maps, were generated (**Figure 2**). **Figure 3** shows the population-based whole-brain fiber tracts transformed from individual space, with each color representing fibers from each individual. The top row shows the fibers from a single subject in the template space. In general, the whole brain fibers from different subjects were overlapped in the template space. **Figure 4** shows the merged tracts overlaid with the DWI metrics on each fiber. Consistent with the DWI metric maps, FA values on the fiber tracts were higher in the regions with highly aligned structures, such as the CC, fornix, and trigeminal nerve. On the contrary, AD, RD and MD values were higher in the regions with more fluid, such as olfactory bulbs, ventricles and cisterns.

### ROI-Based Tracts

**FIGURE 5:**
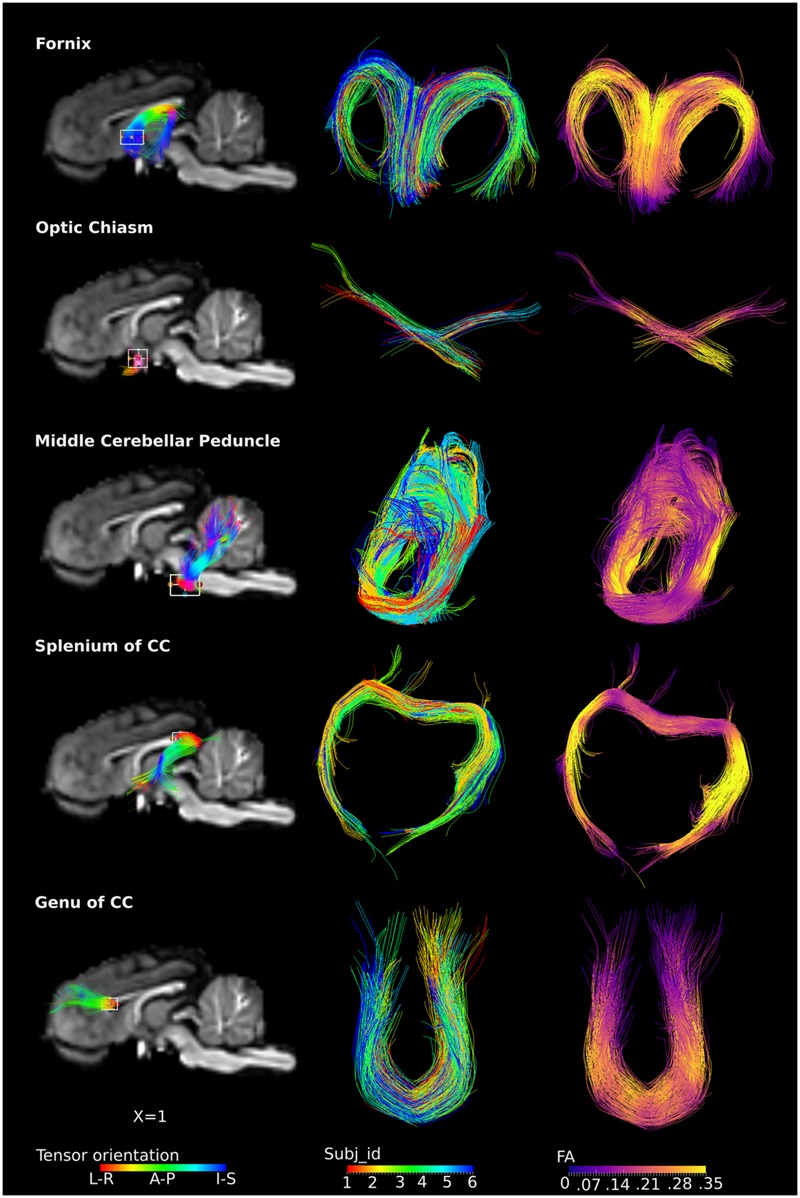
**ROI-specific fibers from fornix, optic chiasm, middle cerebellar peduncle, and splenium and genu of CC.** Column 1 shows the location of the ROIs with a rectangular box, the overlaid fiber tracts are colored by the tensor orientation. Column 2 shows the extracted fibers, with each color representing fibers from each individual. Column 3 shows the extracted fibers colored by the FA values, with brighter color representing higher FA values.

The ROI-specific tracts were extracted from the population-based whole-brain tracts, including tracts from the fornix, optic chiasm, middle cerebellar peduncle, genu and splenium of the CC (**Figure 5**). Column 1 and 3 represent fibers colored by the direction of the tensor and FA values, separately. Column 2 represents fibers colored by individual identities, showing that the tracts from restricted ROIs were consistently present across subjects. Specifically, the fornix could be visualized as the C-shaped fiber bundle from its body, approximately at the level of the anterior commissure, and reaching to the level of the crura. The crossing fibers of the optic chiasm were clearly visualized. The middle cerebellar peduncle fibers were visualized with projections deep into the cerebellum. Similarly, the genu of the CC projections to the bilateral dorsolateral prefrontal region were also seen, with fibers visualized distally and approaching the cortex. For the splenium, the majority of the tracts traveled along the external boundary of lateral ventricles, bilaterally to the temporal lobe (tapetum of the CC), while few tracts traveled to the visual cortex (forceps major).

This template images and tractography are available online^4^, and can be visualized in 3D Slicer.

## Discussion

In this study, we present the first multi-modal piglet brain structural MR template. This template shares the same coordinate origin with existing piglet templates, thus allows for compatibility of the results based on different templates. The T1 template includes tissue segmentation probability maps, which allow for single image segmentation as *a priori* input. The DWI metric template, in the same space as the T1 template, includes FA, AD, RD, and MD maps. These maps make feasible the possibility for gathering a wealth of information on the integrity of WM that is otherwise not available in a T1 template. Over the tracts explored through specific ROIs, similarity of fiber arrangements between piglets and humans are found in the fornix, optic chiasm, the genu of the CC and middle cerebellar peduncles, while different shape is found in the splenium of the CC. The availability of population-based whole-brain fiber tracts allows visualization of the white matter connections at the group level, exploration of specific connections of interest, estimation of their reliability based on the inter-subject consistency, as well as guidance for specific ROI delineation. Additional data from new studies could also be added to this existing population-based fiber tracts to augment the tracts, and thus improve the confidence of the population-wise fiber distribution.

### T1 Template

Compared to the existing two piglet T1 templates, this T1 template includes higher GM/WM contrast retaining more detailed information than the one from Conrad et al. (2014) and is more comprehensive, including both the optic chiasm and olfactory bulb, relative to the one generated by Gan et al. (2014).

A standard coordinate system is important for brain research, where reported brain locations could be easily compared across studies and imaging modalities. In order to maintain consistency with other templates (Felix et al., 1999; Watanabe et al., 2001; Saikali et al., 2010; Conrad et al., 2014; Gan et al., 2014), we set the same origin at the anterior limit of the posterior commissure in the midsagittal plane. This eases the comparison of results from studies with different templates. The validation results presented show only a small deviation (≤1 voxel) between the template and each individual brains. Probability maps of segmentation are also presented here to serve as *a priori* input to aid future single image segmentation.

### DWI Template

Diffusion templates introduced for humans (Mori et al., 2008; Verhoeven et al., 2010; Wang et al., 2011), primates (Adluru et al., 2012; Zakszewski et al., 2014) and rats (Rumple et al., 2013), have played an important role in subsequent ROI-delineation and brain registration (Zhang et al., 2010; Turken and Dronkers, 2011; Vetreno et al., 2015). To date there has been no DWI metric template introduced for the piglet brain, which limits any significant level of information about WM anatomy. In this study, the DWI metric template was generated based on six piglets, providing voxel-wise diffusion information. The different scalar maps could be used as a template for brain normalization. Volumetric-based morphometry analysis of a DWI metric map can also be accomplished with this template.

In this study, with the conjunction uses of HARDI and the method proposed by Tournier et al. (2007) to determine the FOD, orientations that are separated by small angles could be resolved. The fiber tract model thus could represent crossing fibers reliably, e.g., the optic chiasm (**Figure 5**). The population-based whole-brain fiber tracts were generated through transformation of individual streamlines into the template space. These tracts thus represent homologous white matter connections between individual animals. The tracts connecting the same regions are highly overlapped across individuals, reflecting consistency of tracts across subjects (**Figures 3** and **5**), and suggesting that the inter-subject template registrations are within an acceptable range. On the contrary, tracts that show less overlaps may indicate higher structural variability across subjects or lower consistency from tracts generation. Merged tracts are different from tracts generated based on an average diffusion template (Verhoeven et al., 2010; Adluru et al., 2012; Zakszewski et al., 2014), where individual subject diffusion orientation information would have been lost. Furthermore, as the population-based tracts preserve individual tracts information, researchers are able to merge tracts from their own data to the population-based tracts. With the augmented fiber tracts from more subjects, the degree of consistency of the population-wise fiber distribution may be visualized and inspected more confidently.

Specific ROI analysis shows that fiber morphology is analogous in piglets to humans in the fornix (Chen et al., 2015; Kehoe et al., 2015), optic chiasm (Hofer et al., 2010), the genu of the CC (Verde et al., 2014) and middle cerebellar peduncles (Chanraud et al., 2009). However, the shape of the fibers passing through the splenium of the CC is different in piglets and humans. The majority of the splenium fibers project to the visual cortex (called forceps major) while other fibers were part of the tapetum that extended laterally into the temporal lobe in adult human brains (Hofer et al., 2010; Miller et al., 2011; Turken and Dronkers, 2011) as well as neonates (Gilmore et al., 2007; De Bruïne et al., 2011). However, in the piglets, the majority of these fibers form the tapetum, with only a small portion of the tracts projecting to the visual cortex (**Figure 5**). The discrepancy is expected as many brain structural differences exist between the two species (Sauleau et al., 2009). For example, the telencephalon of the pig brain is less curved than that of the human brain (Sauleau et al., 2009).

### Subject Age and Size

The newborn human brain is approximately 36% the size of the adult human brain. With a fast growth speed, the TBV reaches ∼72% of adult brain size at 1 year, and ∼83% at 2 years (Knickmeyer et al., 2008). A similar developmental curve until the maximum is present in the pigs. It is suggested that the piglets reached its maximum brain growth rate at around week 4, when the TBV reached 50% of its maximum, and reached 95% of its maximum by 21–23 weeks of age (Conrad et al., 2012). The age of our piglets is between 17 and 35 days, with an average of 22 days old. The brain size of our piglets is similar to that of the piglets at around 2–5 weeks reported by Conrad et al. (2012). Thus, our template represents the brain with a total volume at around 50% of its maximum brain volume.

### Limitations

The major limitation is that we have a small number of subjects to generate the population-averaged template. However, it has been previously reported that a template stabilizes at around 10 subjects for most human populations based on observations (Avants et al., 2011a). As one of the ungulates, piglets show less complicated convolutional pattern of the brain than humans (Turner, 1890; Welker, 1990; Roth and Dicke, 2005), this eight-subject-based template should therefore be a good representation of brain morphology and its variability of the normal population at this weight range. The mean squared difference comparison between 8-subject-based-template and 2-, 4-, 6-, 8-subject-based templates showed that the templates perceive small differences between the 6-subject-based and 8-subject-based templates (Supplementary Material, Table S1 and Figure S1). Thus, although the sample size is small, the 6-subject-based DWI metric template can serve as a template for the normal piglets at this weight range. Furthermore, the fiber tracts were merged instead of averaged across subjects, extra number of subjects would not influence existing fiber locations or shape. Researchers can augment the tracts by merging tracts from their own data to the population-based tracts. To our knowledge, our atlas template study is the first study investigating the white matter connections of the piglet brain. The population-based fiber tracts presented here will facilitate ROI delineation study of the piglet brain.

## Author Contributions

All authors contributed to this work. The ideas of the manuscript were discussed with the whole project team. JZ, MW, AW, KP, and TL collected the data. JZ wrote the manuscript. DC, AW, KP, JD, MW, and MH commented on the manuscript and gave conceptual advice at the final stage.

## Conflict of Interest Statement

The authors declare that the research was conducted in the absence of any commercial or financial relationships that could be construed as a potential conflict of interest.

## Footnotes

1 https://www.slicer.org/

2 http://fsl.fmrib.ox.ac.uk/fsl/fslwiki/

3 http://www.brain.org.au/software/

4 http://www.hodaielab.com/resources.html
