# Supplementary material for: An In vivo Multi-Modal Structural Template for Neonatal Piglets Using High Angular Resolution and Population-Based Whole-Brain Tractography
Source: Front Neuroanat. 2016 Sep 27;10:92. doi: 10.3389/fnana.2016.00092 (PMC5037218; doi:10.3389/fnana.2016.00092)
Supplement: Supplementary file 1 [file Data_Sheet_1.PDF]

Supplementary Material

Template distance calculation

To investigate whether template generated based on 8 subjects ($Temp_8$) could represent brain morphology and its variability of the piglets weighed around 6 kg, we additionally generated templates based on 2, 4, and 6 subjects ($Temp_2$, $Temp_4$, and $Temp_6$). The three templates were automatically registered to the $Temp_8$ with affine registration using ANTs (Figure S1). Then, the mean squared difference between the additional templates and $Temp_8$ were calculated. To remove the effect of interpolation during registration, we generated an affine-transformed $Temp_8$ for $Temp_8$ with a random transformation to serve as the ground truth ($Temp_{8\text{-sim}}$). This template was also registered to $Temp_8$ with affine registration and the mean squared difference was calculated. It can be seen that the difference between $Temp_6$ and $Temp_8$ were smaller than that for $Temp_2$ and $Temp_4$, but similar to that between $Temp_{8\text{-sim}}$ and $Temp_8$, which is the ground truth (Table S1). The result suggests that $Temp_6$ and $Temp_8$ are similar in shape and $Temp_6$ could be representative enough to the population average.

Table S1. Mean squared difference between templates generated based on different number of subjects and our 8-subject-based template

	$Temp_2$	$Temp_4$	$Temp_6$	$Temp_{8\text{-sim}}$
Mean squared difference, mm	0.238	0.159	0.134	0.135

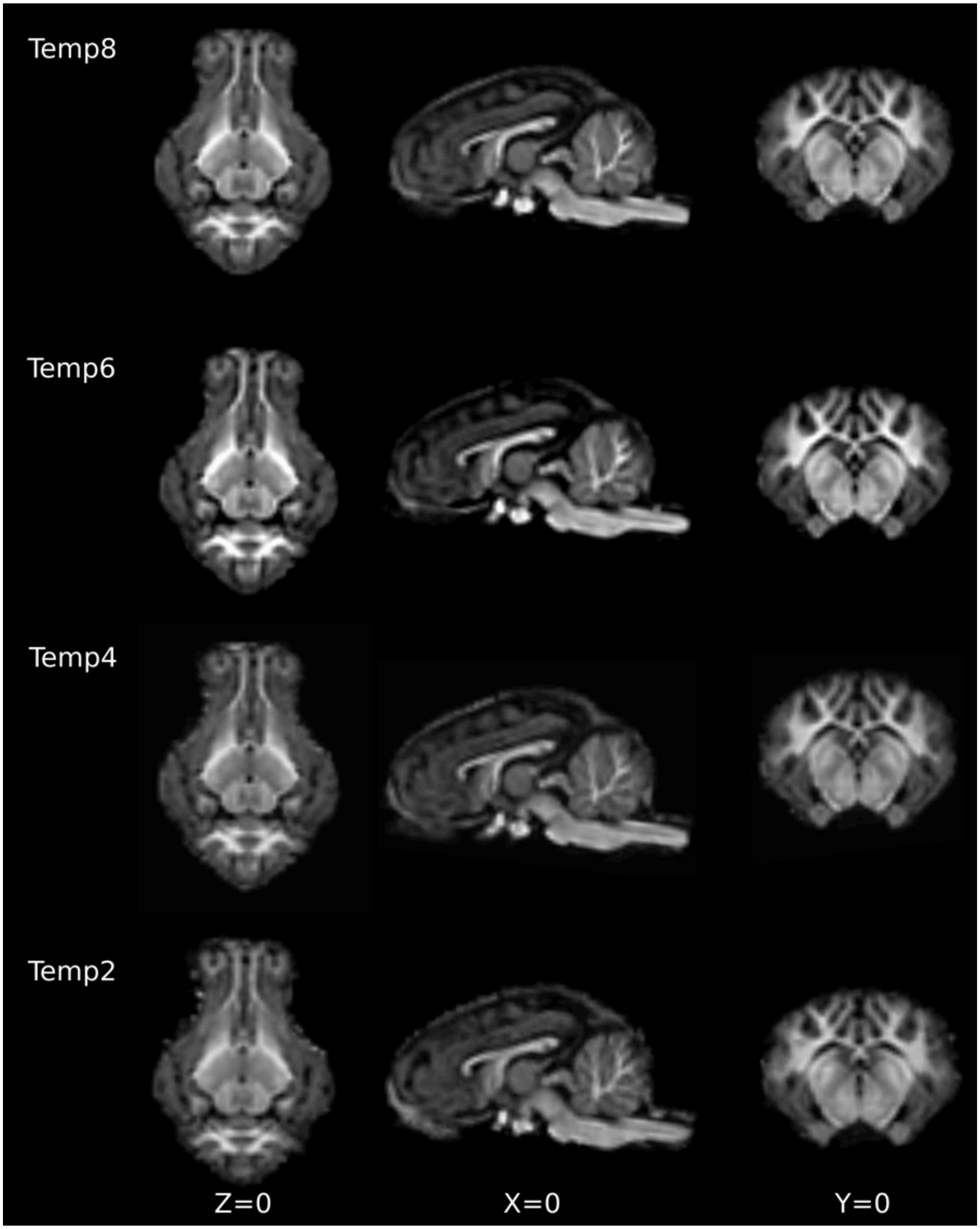

Figure S1. Templates generated based on different number of subjects are shown in the axial (Z=0), sagittal (X=0) and coronal (Y=0) views. Templates generated based on 6 and 8 subjects present similar WM and GM features.
